# Diagnostic accuracy of ePOS score in predicting DNR labeling after ICU admission: A prospective observational study (ePOS-DNR)

**DOI:** 10.1016/j.jointm.2023.09.003

**Published:** 2023-11-04

**Authors:** Omar E. Ramadan, Ahmed F. Mady, Mohammed A. Al-Odat, Ahmed N. Balshi, Ahmed W. Aletreby, Taisy J. Stephen, Sheena R. Diolaso, Jennifer Q. Gano, Waleed Th. Aletreby

**Affiliations:** 1 Department of Critical Care, King Saud Medical City, Riyadh, Saudi Arabia; 2 Anesthesia Department, Faculty of Medicine, Ain Shams University, Cairo, Egypt; 3 Anesthesia Department, Faculty of Medicine, Tanta University, Tanta, Egypt; 4 Faculty of Medicine, Alexandria University, Alexandria, Egypt; 5 Nursing Department, King Saud Medical City, Riyadh, Saudi Arabia

**Keywords:** Do-not-resuscitate (DNR), Resuscitation, Intensive care unit (ICU), Diagnostic accuracy, Sensitivity, Specificity

## Abstract

**Background:**

Resuscitation can sometimes be futile and making a do-not-resuscitate (DNR) decision is in the best interest of the patient. The electronic poor outcome screening (ePOS) score was developed to predict 6-month poor outcomes of critically ill patients. We explored the diagnostic accuracy of the ePOS score in predicting DNR decisions in the intensive care unit (ICU).

**Methods:**

This study was conducted at the ICU of a tertiary referral hospital in Saudi Arabia between March and May 2023. Prospectively, we calculated ePOS scores for all eligible consecutive admissions after 48 h in the ICU and recorded the DNR orders. The ability of the score to predict DNR was explored using logistic regression. Youden's ideal cut-off value was calculated using the DeLong method, and different diagnostic accuracy measures were generated with corresponding 95 % confidence intervals (CIs).

**Results:**

We enrolled 857 patients, 125 received a DNR order and 732 did not. The average ePOS score of DNR and non-DNR patients was 28.2±10.7 and 15.2±9.7, respectively. ePOS score, as a predictor of DNR order, had an area under receiver operator characteristic (AUROC) curve of 81.8 % (95% CI: 79.0 to 84.3, *P* <0.001). Youden's ideal cut-off value >17 was associated with a sensitivity of 87.2 (95% CI: 80.0 to 92.5, *P* <0.001), specificity of 63.9 (95% CI: 60.3 to 67.4, *P* <0.001), positive predictive value of 29.2 (95% CI: 24.6 to 33.8, *P* <0.001), negative predictive value of 96.7 (95% CI: 95.1 to 98.3, *P* <0.001), and diagnostic odds ratio 12.1 (95% CI: 7.0 to 20.8, *P* <0.001).

**Conclusions:**

In this study, the ePOS score performed well as a diagnostic test for patients who will be labeled as DNR during their ICU stay. A cut-off score >17 may help guide clinical decisions to withhold or commence resuscitative measures.

## Introduction

Advances in medical knowledge, therapy, and technology have resulted in a significant proportion of elderly patients being admitted to the intensive care unit (ICU) with complex multiple comorbidities.^[^^1^^]^ Intensivists find themselves divided between stabilizing, resuscitating, and treating critically ill patients, on the one hand,^[^^2^^,^^3^^]^ and unduly prolonging life, along with patients’ suffering and families’ languishing, on the other hand.^[^^4^^,^^5^^]^ All the while, they must use already scarce ICU resources efficiently.^[^^4^^,^^6^^]^ Since the introduction of cardiopulmonary resuscitation (CPR) in the 1960s,^[^^7^^]^ it has become clear that CPR is not necessarily beneficial for all patients, given their condition and the futility of treatment,^[^^8^^,^^9^^]^ Accordingly, the concept of do-not-resuscitate (DNR) has become widely accepted in practice since the 1970s^[^^9^^]^ to avoid the harms of futile aggressive resuscitation in a specific group of patients.^[^^2^^]^

Identifying patients for whom a DNR decision would be in their best interest is difficult, complex, and challenging,^[^^1^^,^^2^^]^ mainly because this decision must be made promptly to avoid the aforementioned harm. However, a high percentage of DNR orders are made shortly before death, when suffering has already occurred and resources have been wasted.^[^^10^^,^^11^^]^ Additionally, there is no consensus on medical futility^[^^12^^]^ or guidance on the practical aspects of the DNR process, such as objective criteria.^[^^13^^]^

Luethi et al.^[^^1^^]^ developed the “electronic poor outcome screening (ePOS)” score to predict the risk of 6-month all-cause mortality in critically ill patients. The score is easy to calculate, uses readily available data, and is calculated after 48 h in the ICU. The score showed good discriminatory power, with an area under receiver operator characteristic (AUROC) curve of 72% (95% confidence interval [CI]: 67 to 77). They concluded that it can identify patients at high risk of death within 6 months so that palliative services can be provided.

We hypothesized that the ePOS score could also predict, with reasonable diagnostic accuracy, patients being issued DNR orders during their ICU stay. This study investigated the diagnostic accuracy of the ePOS score in predicting DNR orders in the ICU and its ability to guide DNR decision-making without replacing the clinical judgment of intensivists.

## Methods

### Design, setting, and timeframe

This prospective observational study was conducted in the ICU of a large tertiary referral hospital in the central region of Saudi Arabia. Our hospital has 1200 inpatient beds, and the ICU houses 110 beds fully equipped with facilities for invasive and non-invasive mechanical ventilation and monitoring. It is a closed ICU, staffed round the clock by intensivists, with a nurse-to-patient ratio of 1:1. The study was conducted prospectively for 3 months between March 1, 2023, and May 31, 2023. Follow-up continued until the hospital outcome of the last included patient was known. We followed the Standards for Reporting Diagnostic Accuracy Studies (STARD) checklist for this report.^[^^14^^]^

### DNR procedure

In our ICU, we follow the National Policy of DNR issued by the Saudi Health Council.^[^^15^^]^ The treating consultant identifies a patient for whom further aggressive management is expected to be futile and refers the patient to two other colleagues (at least specialists). The two other physicians review the patient independently, and if a consensus is reached, three of them will sign the DNR form. While making the DNR decision, in addition to refraining from CPR in case of cardiac arrest, one of the three categories must be clearly indicated: DNR with withholding (no further aggressive management modalities are added to the current ones); DNR with withdrawal (current treatment modalities can be withdrawn); and DNR with limited escalation (management can still be escalated using certain predefined interventions). The national DNR policy also defines the procedure of DNR reversal and encourages family involvement in the decision, although it is not mandatory.

### Inclusion and exclusion criteria

We included all adult patients (age ≥18 years) admitted to the ICU during the study period. We excluded patients who were admitted to the ICU with an advance directive or who were discharged from the ICU before 48 h because they died, were transferred to another hospital, or had a rapid recovery (such as patients admitted for brief postoperative monitoring). To maintain data independence, we also excluded any subsequent readmissions of an already included patient (whether or not the readmission occurred within the same hospitalization).

### Calculation of ePOS score and data management

The score includes 11 categorical variables. Each variable was assigned a score, and the final ePOS score was the sum of all scores. Seven variables were recorded at ICU admission, while four variables were recorded 48 h after ICU admission. The score ranged from 0 to 70 points (Supplementary Table S1).

During the study period, three authors (TS, SD, and JG) identified new ICU admissions, verified their eligibility, and recorded the seven admission variables. After 48 h, the remaining four variables were recorded if the patient was still in the ICU. Patients with completed scoring variables were followed up for ICU and hospital outcomes. Patients transferred to other hospitals were considered discharged alive from our hospital and were not followed up. The issuance of a DNR order by the attending physician was recorded for each included patient and was not limited to a specific time. All data were pseudonymized and entered into a predesigned online sheet, and four authors (AA, OR, AM, and WA) scored the variables for each patient without knowing whether a DNR order was issued. Treating team members were blinded to the ePOS score. All authors who collected and scored the ePOS variables were well-trained and familiar with the scoring method.

### Study outcomes

The primary outcome was the diagnostic accuracy measures of the ePOS score in discriminating DNR patients. This included the AUROC curve as well as the ideal criterion (cut-off value) and its associated sensitivity, specificity, positive predictive value (PPV), negative predictive value (NPV), positive likelihood ratio (LR+), negative likelihood ratio (LR−), and diagnostic odds ratio (DOR). Details of calculations are provided in Supplementary Table S2.

The secondary outcomes included demographic and clinical comparisons between DNR and non-DNR patients by age, gender, ePOS score, ICU length of stay (LOS), hospital LOS, ICU disposition, and hospital all-cause mortality.

#### Minimum required sample

We estimated that a minimum of 541 patients (including 81 with DNR orders) was required to significantly detect an AUROC of 75% (compared to a null of 65%) with a power of at least 90% and a type I error rate of 5%. According to a previous study conducted in our ICU, we assumed a prevalence of DNR in ICU-discharged patients of 15%.^[^^13^^]^

#### Statistical considerations

The primary outcome of diagnostic accuracy was evaluated using the DeLong method^[^^16^^]^ of AUROC curve evaluation. The ePOS score was used as a continuous variable, while the classification variable was the binary outcome of DNR or not. A calibration belt was constructed from an univariable logistic regression model, with DNR as the dependent and ePOS score as the independent variable. The calibration belt was presented within 95% and 99% CIs. All components of diagnostic accuracy were presented for Youden's index J (sensitivity + specificity − 1) and its associated criterion, along with the corresponding 95% CI, calculated using bootstrap with 1000 iterations.

For the secondary outcomes, categorical variables were summarized as frequency and percentage (%) and compared between groups using Pearson's chi-squared test or Fisher's exact test as appropriate. Continuous variables were summarized as mean±standard deviation (SD) and compared between groups using the Student's *t*-test or Wilcoxon rank sum test as appropriate.

All tests were considered statistically significant if the *P*-value was <0.05 without correction for multiple testing. As such, results of secondary outcomes should not be considered conclusive. All tests were performed using the commercially available statistical software Medcalc® (MedCalc for Windows, version 19.2.6 [MedCalc Software, Ostend, Belgium]).

### Ethical considerations

We obtained the approval of the local institutional review board (H1RI-31-Jan23–01), with a waiver of consent due to its observational nature and lack of intervention by the study team. We applied all the research subjects’ protection principles outlined by the Declaration of Helsinki. We prospectively registered the study protocol (UIN: researchregistry8672).

## Results

**Figure 1:**
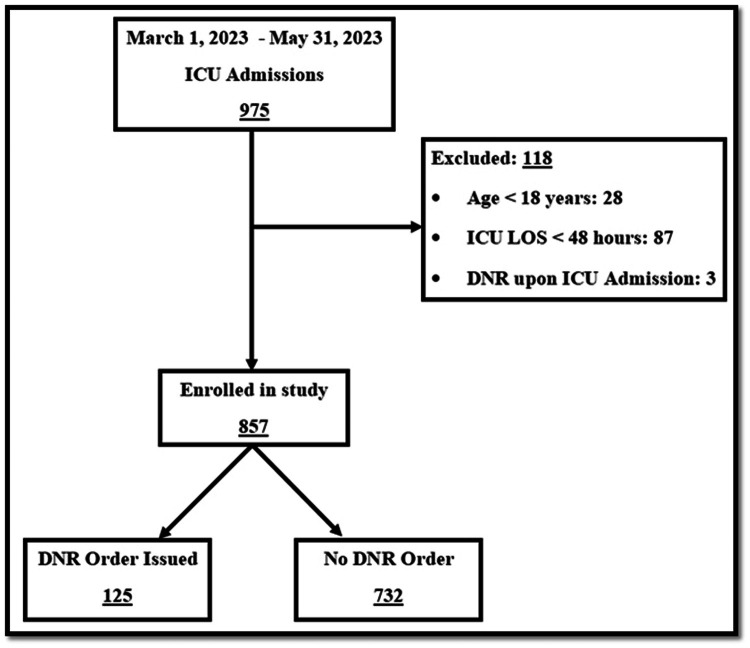
Flow diagram of patient enrollment. DNR: Do not resuscitate; ICU: Intensive care unit; LOS: Length of stay.

During the study period, there were 975 admissions to the ICU. We excluded 118 patients and enrolled 857 in the study. A total of 125 (14.6%) patients were issued with DNR orders during their ICU stay, and 732 (85.4%) patients were discharged from the ICU without DNR orders (Figure 1).

The DNR patients were significantly older than non-DNR, but the gender distribution was similar. DNR patients had a significantly higher APACHE Ⅳ score and were more frequently mechanically ventilated upon ICU admission. The most common admission diagnosis among DNR patients was septic shock, followed by ischemic cerebrovascular stroke. The most common diagnosis for non-DNR patients was trauma, followed by acute respiratory distress syndrome. Most DNR patients (95.2%) and 54.8% of non-DNR patients were mechanically ventilated upon ICU admission. The mean ePOS score of the DNR group was significantly higher than the non-DNR group (DNR: 28.2±10.7; non-DNR: 15.2±9.7; 95% CI of difference: −14.9 to −11.1; *P* <0.001). Almost all of the DNR patients (124 [99.2 %]) died in the hospital, and only one patient was discharged home. By contrast, 78 (10.7%) of non-DNR patients died in the hospital. All deaths occurred in the ICU. The ICU LOS of DNR patients was significantly higher than for the non-DNR patients. Hospital LOS, however, was not significantly different between groups, although numerically higher for the DNR group (Table 1).

**Table 1:** Demographic and clinical outcomes comparison between groups.

Variable	All (*n*=857)	DNR (*n*=125)	Non-DNR (*n*=732)	95 % CI of difference	*P*-value
Age (years)	48.9±20.0	60.6±17.4	46.9±19.8	−17.4 to −10.0	<0.001*
Gender (males)	576 (67.2)	81 (64.8)	495 (67.6)	−6.2 to 12.5	0.600
APACHE IV score	68.3±12.2	72.7±13.8	67.5±11.7	2.6 to 7.8	<0.001
Mechanically ventilated upon ICU admission	520 (60.7)	119 (95.2)	401 (54.8)	33.9 to 45.2	<0.001
ICU admission diagnosis					
Ischemic cerebrovascular stroke	113 (13.2)	31 (24.8)	82 (11.2)	5.9 to 22.4	<0.001
Septic shock	169 (19.7)	47 (37.6)	122 (16.7)	11.9 to 30.4	<0.001
ARDS	150 (17.5)	14 (11.2)	136 (18.6)	−0.01 to 13.2	0.060
Cranial bleeding (non-traumatic)	95 (11.1)	17 (13.6)	78 (10.7)	−3.1 to 10.5	0.400
Trauma	193 (22.5)	4 (3.2)	189 (25.8)	−16.9 to −26.7	<0.001
Metabolic disorders	44 (5.1)	0 (0)	44 (6)	−2.7 to −8.0	0.010
COPD exacerbation	47 (5.5)	0 (0)	47 (6.4)	−3.1 to −8.4	0.010
Surgical exploration	34 (4)	0 (0)	34 (4.6)	−1.4 to −6.4	0.030
Metastatic malignancy	12 (1.4)	12 (9.6)	0 (0)	5.0 to 16.2	<0.001
ePOS score	17.1±10.9	28.2±10.7	15.2±9.7	−14.9 to −11.1	<0.001*
ICU LOS (days)	13.3±15.5	18.3±20.8	12.5±14.3	−8.7 to −2.8	0.020*
Hospital LOS (days)	21.2±22.5	24.1±25.7	20.7±21.9	−7.6 to 1.0	0.800*
Hospital mortality	202 (23.6)	124 (99.2)	78 (10.7)	84.2 to 90.8	<0.001*
Hospital disposition					
Discharged alive	580 (67.7)	1 (0.8)	579 (79.1)	73.6 to 81.3	<0.001
Death	202 (23.6)	124 (99.2)	78 (10.7)	84.2 to 90.8	<0.001
Transfer to other hospitals	75 (8.7)	0 (0)	75 (10.2)	6.6 to 12.6	<0.001

Data are expressed as *n* (%), mean±standard deviation.

⁎ Wilcoxon rank sum test. APACHE: Acute Physiology and Chronic Health Evaluation; ARDS: Acute respiratory distress syndrome; CI: Confidence interval; COPD: Chronic obstructive pulmonary disease; DNR: Do not resuscitate; ePOS: Electronic poor outcome screening; ICU: Intensive care unit; LOS: Length of stay.

Notably, 76.8% of the DNR orders were issued after 48 h of ICU admission, and 63.2% were of the withdrawal category (Supplementary Table S3).

### Primary outcomes

**Figure 2:**
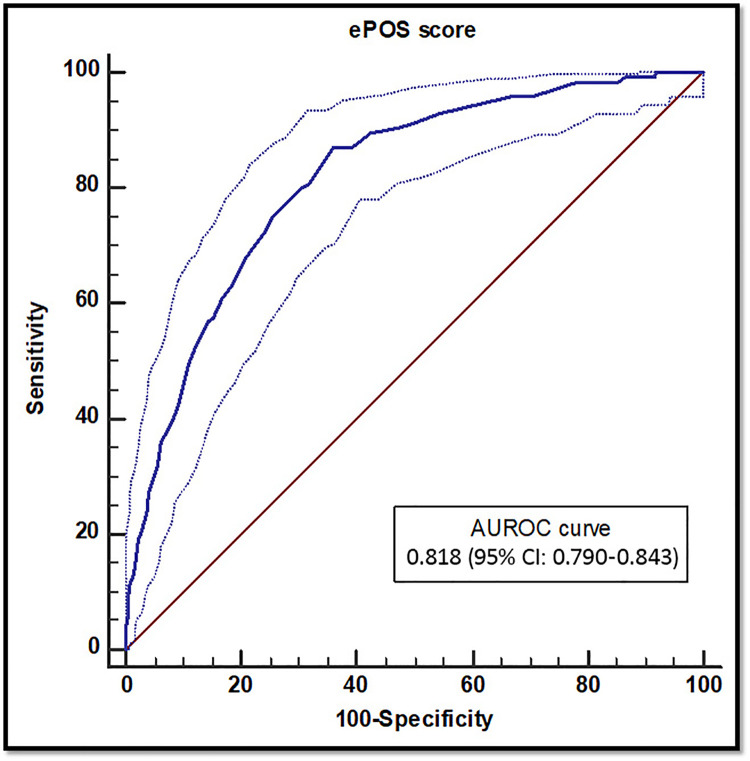
AUROC curve for diagnostic accuracy. AUROC: Area under receiver operator characteristic; CI: Confidence interval.

**Figure 3:**
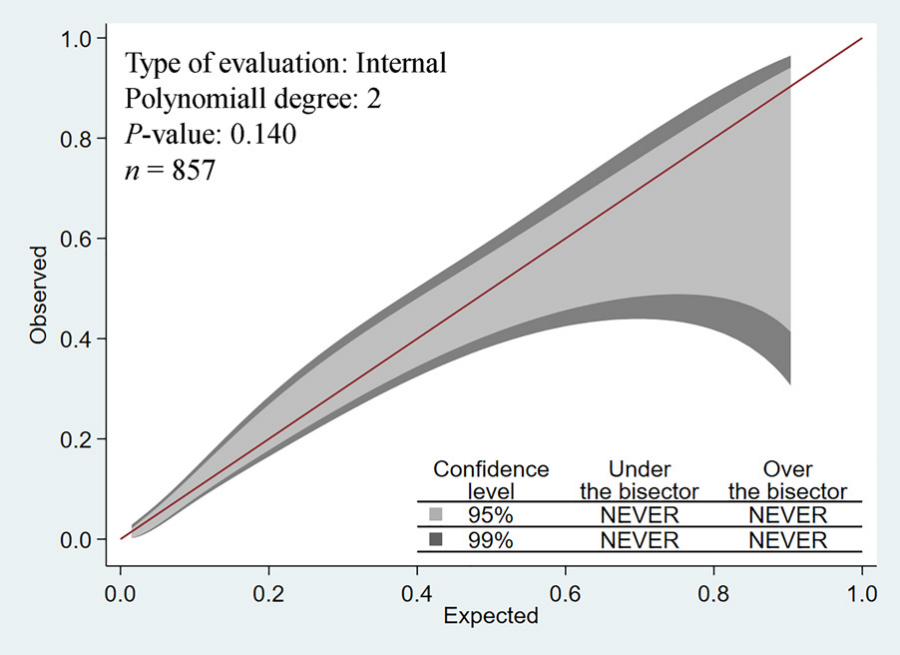
Calibration belt for electronic poor outcome screening score prediction.

The diagnostic accuracy analysis of the ePOS score showed an AUROC curve of 81.8% (95% CI: 79.0 to 84.3, *P* <0.001) (Figure 2). The calibration belt of prediction was not statistically different from the perfect prediction (*P*=0.100) within the 95% and 99% CIs (Figure 3). Youden's index was 0.51, and its associated criterion was >17. Using this cut-off value yielded the diagnostic accuracy measures presented in Table 2.

**Table 2:** Diagnostic accuracy measure for ePOS cut-off value >17.

Diagnostic accuracy measure	Value	95 % CI	*P*-value
AUROC curve	81.8	79.0 to 84.3	<0.001
Sensitivity (%)	87.2	80.0 to 92.5	
Specificity (%)	63.9	60.3 to 67.4	
PPV (%)	29.2	24.6 to 33.8	
NPV (%)	96.7	95.1 to 98.3	
LR+ (%)	2.4	2.1 to 2.7	
LR− (%)	0.2	0.1 to 0.3	
DOR (%)	12.1	7.0 to 20.8	

AUROC: Area under receiver operator characteristic; CI: Confidence interval; DOR: Diagnostic odds ratio; LR−: Negative likelihood ratio; LR+: Positive likelihood ratio; NPV: Negative predictive value; PPV: Positive predictive value.

Supplementary Table S4 shows sensitivity and specificity combinations of all cut-off values. Supplementary Table S5 shows the crude values from which diagnostic accuracy measures were calculated for the cut-off value of >17. The cut-off value of >17 correctly classified 109 of 125 true positive cases and 468 of 732 true negative cases.

## Discussion

The ePOS score had a very good^[^^17^^]^ AUROC curve in this study. It performed well in discriminating between DNR and non-DNR patients by correctly classifying 109 of 125 true positives (87.2%); it also had high predictive power, especially NPV.

The various measures of diagnostic accuracy are not fixed measures of the quality of a test but are rather influenced by the characteristics of the study population. Nevertheless, they are of considerable clinical importance to clinicians when their different roles and functions are understood.^[^^18^^]^ The AUROC curve is a widely used measure of the accuracy of a test and reflects how high its discriminatory power is.^[^^17^^]^ Sensitivity and specificity are specific discriminatory assessment properties. Sensitivity reflects the probability of a positive test in the presence of the disease and refers to the test's potential in detecting patients with the disease or condition.^[^^17^^]^ We found that the probability of a result >17 was 87.2% if the patient was labeled as DNR. Specificity refers to the ability of the test to detect patients without the disease, and the probability of a score ≤17 was 63.9% if the patient was not labeled as DNR. Perhaps more important to clinicians are the predictive abilities of the test represented by PPV and NPV. The NPV was strikingly high in the current study, meaning that the probability of not labeling the patient as DNR was 96.7% when the score was ≤17.^[^^17^^]^ This is clinically important because it gives physicians a rough guide to providing resuscitative measures to patients with a score of ≤17. The PPV, however, does not give a strong indication to designate the patient as DNR when the score is >17 because the probability of DNR in this case is only 29.2%. Both predictive values in this study should not be generalized to populations because they are both strongly related to DNR prevalence in the study.^[^^17^^,^^18^^]^ However, NPV may increase in a population with a lower prevalence of DNR,^[^^17^^]^ and in this study, NPV appears to be the most valuable measure of diagnostic accuracy because it identifies patients for whom resuscitation can reasonably result in survival outside of acute care setting, and limits consideration of CPR as “futile” to circumstances in which it cannot achieve its intended goal.^[^^19^^]^ The positive and negative likelihood ratios were consistent with expectations for a test with good discriminatory power. The LR+ was greater than 1, indicating a higher likelihood of a score >17 for DNR patients compared with non-DNR patients. LR− below one also indicates a lower probability of a negative test (score ≤17) for DNR patients compared with non-DNR patients. However, none of the likelihood ratios reached the limits commonly accepted to rule in or out the disease (LR+ >10 and LR− <0.1).^[^^17^^]^ A DOR of approximately 12 in our study also suggests good discriminatory ability of the test as a single accuracy measure combining the strength of sensitivity and specificity without being affected by prevalence.^[^^20^^]^

Several attempts have been made to develop scores with specific cut-off points to identify patients for whom CPR may be considered futile. Scores such as the “pre-arrest morbidity” (PAM) index and the “prognosis after resuscitation” score did not adequately differentiate between survivors and non-survivors after resuscitation,^[^^21^^]^ with AUROC curves of 74% and 67%, respectively.^[^^22^^]^ A more promising score was the “good outcome following attempted resuscitation” (GO-FAR) score, which had an AUROC curve of 82% ^[^^23^^]^ and a higher value of 85% in a validation study.^[^^24^^]^ Although the GO-FAR score has the advantage of predicting survival with intact neurologic function after resuscitation, its calibration plots showed that it systematically underestimated this probability. This underestimation motivated a modification of the model into “Pre-arrest prediction of outcome after In Hospital Cardiac Arrest” (PIHCA), which still lacks external validation for populations other than the Swedish population for which it was developed; it also requires multiple laboratory tests for its calculation that may not always be readily available.^[^^25^^]^

To the best of our knowledge, this is the first study to examine the diagnostic accuracy of the ePOS score in identifying patients who are labeled DNR during their ICU stay. The score performed well, especially in excluding DNR status with a score of ≤17. Accordingly, it could be a good objective tool to aid the clinical judgment of intensivists trying to identify critically ill patients in whom resuscitation may or may not be beneficial.

### Limitations

Our study is subject to several limitations in addition to the inherent limitations of the observational design. Although it is sufficiently powered for its primary outcome, it has a relatively small sample size when considered as a validation study. The predictive power of the score (PPV and NPV) in our study cannot be generalized to other populations with different DNR prevalence, and we strongly recommend external validation studies. We could not follow up on patients transferred to other hospitals to determine whether any of them later received a DNR order. The analysis was conducted on DNR patients without differentiating between the different categories of DNR orders due to the small number of each category. Although we blinded the treating team to the ePOS score, they were aware of the study's title; some may have been familiar with the score and calculated it themselves. In this case, we cannot be sure whether or not this biased their decision to issue a DNR order. Finally, perhaps more complex statistical models incorporating other patients’ characteristics could have been used to explore the primary outcome. Still, those models would have also been difficult to interpret, precluding the clinical importance of the study.

## Conclusions

In this study, the ePOS score performed well as a diagnostic test for patients who will be labeled as DNR during their ICU stay. A cut-off score >17 may help guide decisions to withhold or commence resuscitative measures.

## Author Contributions

**Taisy J. Stephen, Sheena R. Diolaso**, and **Jennifer Q. Gano:** Eligibility check, data recording, and follow-up. **Ahmed W. Aletreby, Omar E. Ramadan, Ahmed F. Mady**, and **Waleed Th. Aletreby:** ePOS score calculation. **Omar E. Ramadan, Ahmed F. Mady**, and **Mohammed A. Al-Odat:** Concept and design. **Ahmed N. Balshi** and **Ahmed W. Aletreby:** Literature review. **Waleed Th. Aletreby:** Formal analysis and visualization. **Omar E. Ramadan, Ahmed F. Mady, Mohammed A. Al-Odat, Ahmed N. Balshi**, and **Waleed Th. Aletreby**: Drafting the manuscript. All authors: Revision and approval of final manuscript.

## Acknowledgments

None.

## Funding

This research did not receive any specific grant from funding agencies in the public, commercial, or not-for-profit sectors.

## Ethics Statement

The study is approved by the local IRB (H1RI-31-Jan23-01). Registered at researchregistry.com (UIN: researchregistry8672). Registered protocol can be accessed at: https://www.researchregistry.com/browse-the-registry#home/registrationdetails/63e8547e9b4a130011ee62d1/.

## Conflict of Interest

The authors declare that they have no known competing financial interests or personal relationships that could have appeared to influence the work reported in this paper.

## Data Availability

The data sets generated during and/or analyzed during the current study are available from the corresponding author upon reasonable request.

## Appendix Supplementary materials

Image, application 1
