# Supplementary material for: Diagnostic accuracy of ePOS score in predicting DNR labeling after ICU admission: A prospective observational study (ePOS-DNR)
Source: J Intensive Med. 2023 Nov 4;4(2):216–21. doi: 10.1016/j.jointm.2023.09.003 (PMC11043627; doi:10.1016/j.jointm.2023.09.003)
Supplement: Supplementary file 1 [file mmc1.docx]

**Supplementary file:**

**Table S1: ePOS score calculation**

**Table S2: A: 2 X 2 contingency table**

**B: Diagnostic accuracy measures calculation.**

**Table S3: Timing and categories of DNR orders.**

**Table S4: Diagnostic accuracy of all cutoff points.**

**Table S5: DNR classification of >17 cutoff value.**

**Table S1: ePOS score calculation:**

| Variable | Categories | score |
| --- | --- | --- |
| Age Category | < 60 years  60 to 69 years  70 to 79 years  > 79 years | 0  5  9  14 |
| Functional Status | Independent  Fully or partially dependent in at least one daily activity | 0  3 |
| Malignancy | None  Active malignancy | 0  3 |
| Number of hospitalizations within last 6 months | None  1-2  More than 2 | 0  2  5 |
| Duration between hospital and ICU admission | Less than 24 hours  More than 24 hours | 0  11 |
| Type of ICU admission | Surgical (elective/emergency)  Medical | 0  4 |
| ICU admission diagnosis | Cardiovascular disease/Respiratory failure  Non-traumatic cerebral pathology  Cardiac arrest  Other | 2  7  7  0 |
| Serum Lactate > 2 mmol/L  (at 48 hours of ICU admission) | YES  NO | 7  0 |
| Invasive Mechanical Ventilation  (at 48 hours of ICU admission) | YES  NO | 9  0 |
| Vasoactive Drugs  (at 48 hours of ICU admission) | YES  NO | 2  0 |
| Continuous Renal Replacement Therapy  (at 48 hours of ICU admission) | Yes  NO | 5  0 |

Luethi N, Wermelinger SD, Haynes AG, Roumet M, Maessen M, Affolter B, Müller M, Schefold JC, Eychmueller S, Cioccari L. Development of an electronic Poor Outcome Screening (ePOS) Score to identify critically ill patients with potential palliative care needs. J Crit Care. 2022 Jun;69:154007.

**Table S2: A: 2 X 2 contingency table**

|  |  | **Actual DNR Status** | |
| --- | --- | --- | --- |
|  |  | **+** | **-** |
| **ePOS Classification** | **+** | **TP** | **FP** |
|  | **-** | **FN** | **TN** |

TP = true positive, TN = true negative, FP = false positive, FN = false negative

**Table S2: B: Diagnostic accuracy measures calculation.**

| **Diagnostic Accuracy Measure** | **Calculation** |
| --- | --- |
| **Sensitivity** | **TP / (TP + FN)** |
| **Specificity** | **TN / (TN + FP)** |
| **Positive Predictive Value** | **TP / (TP + FP)** |
| **Negative Predictive Value** | **TN / (TN + FN)** |
| **Positive Likelihood Ratio** | **sensitivity / (1 – specificity)** |
| **Negative Likelihood Ratio** | **(1 – sensitivity) / Specificity** |
| **Diagnostic Odds Ratio** | **(TP/FN)/(FP/TN)** |

**Table S3: Timing and categories of DNR orders:**

| **Timing of DNR** | DNR Patients (n = 125) |
| --- | --- |
| DNR within 48 hours of ICU admission (early DNR) | 29 (23.2%) |
| DNR after 48 hours of ICU admission (Late DNR) | 96 (76.8%) |
| **DNR Category** |  |
| Withholding | 18 (14.4%) |
| Withdrawal | 79 (63.2%) |
| Limited Escalation | 28 (22.4%) |

**Table S4: Diagnostic accuracy of all cutoff points.**

| Criterion | Sensitivity | 95% CI | Specificity | 95% CI | +LR | 95% CI | -LR | 95% CI | +PV | -PV |
| --- | --- | --- | --- | --- | --- | --- | --- | --- | --- | --- |
| ≥0 | 100.00 | 97.1 - 100.0 | 0.00 | 0.0 - 0.5 | 1.00 | 1.0 - 1.0 |  |  | 14.6 |  |
| >0 | 100.00 | 97.1 - 100.0 | 7.92 | 6.1 - 10.1 | 1.09 | 1.1 - 1.1 | 0.00 |  | 15.6 | 100.0 |
| >2 | 99.20 | 95.6 - 100.0 | 8.33 | 6.4 - 10.6 | 1.08 | 1.1 - 1.1 | 0.096 | 0.01 - 0.7 | 15.6 | 98.4 |
| >3 | 99.20 | 95.6 - 100.0 | 9.29 | 7.3 - 11.6 | 1.09 | 1.1 - 1.1 | 0.086 | 0.01 - 0.6 | 15.7 | 98.6 |
| >4 | 99.20 | 95.6 - 100.0 | 13.52 | 11.1 - 16.2 | 1.15 | 1.1 - 1.2 | 0.059 | 0.008 - 0.4 | 16.4 | 99.0 |
| >5 | 98.40 | 94.3 - 99.8 | 14.75 | 12.3 - 17.5 | 1.15 | 1.1 - 1.2 | 0.11 | 0.03 - 0.4 | 16.5 | 98.2 |
| >6 | 98.40 | 94.3 - 99.8 | 16.94 | 14.3 - 19.9 | 1.18 | 1.1 - 1.2 | 0.094 | 0.02 - 0.4 | 16.8 | 98.4 |
| >7 | 98.40 | 94.3 - 99.8 | 20.22 | 17.4 - 23.3 | 1.23 | 1.2 - 1.3 | 0.079 | 0.02 - 0.3 | 17.4 | 98.7 |
| >8 | 98.40 | 94.3 - 99.8 | 21.86 | 18.9 - 25.0 | 1.26 | 1.2 - 1.3 | 0.073 | 0.02 - 0.3 | 17.7 | 98.8 |
| >9 | 96.00 | 90.9 - 98.7 | 29.51 | 26.2 - 33.0 | 1.36 | 1.3 - 1.4 | 0.14 | 0.06 - 0.3 | 18.9 | 97.7 |
| >10 | 96.00 | 90.9 - 98.7 | 33.06 | 29.7 - 36.6 | 1.43 | 1.3 - 1.5 | 0.12 | 0.05 - 0.3 | 19.7 | 98.0 |
| >11 | 93.60 | 87.8 - 97.2 | 43.31 | 39.7 - 47.0 | 1.65 | 1.5 - 1.8 | 0.15 | 0.08 - 0.3 | 22.0 | 97.5 |
| >12 | 92.80 | 86.8 - 96.7 | 45.77 | 42.1 - 49.5 | 1.71 | 1.6 - 1.9 | 0.16 | 0.08 - 0.3 | 22.6 | 97.4 |
| >13 | 91.20 | 84.8 - 95.5 | 50.55 | 46.9 - 54.2 | 1.84 | 1.7 - 2.0 | 0.17 | 0.10 - 0.3 | 23.9 | 97.1 |
| >14 | 90.40 | 83.8 - 94.9 | 52.87 | 49.2 - 56.5 | 1.92 | 1.7 - 2.1 | 0.18 | 0.1 - 0.3 | 24.7 | 97.0 |
| >15 | 89.60 | 82.9 - 94.3 | 57.65 | 54.0 - 61.3 | 2.12 | 1.9 - 2.3 | 0.18 | 0.1 - 0.3 | 26.5 | 97.0 |
| >16 | 87.20 | 80.0 - 92.5 | 60.66 | 57.0 - 64.2 | 2.22 | 2.0 - 2.5 | 0.21 | 0.1 - 0.3 | 27.5 | 96.5 |
| >17 | 87.20 | 80.0 - 92.5 | 63.93 | 60.3 - 67.4 | 2.42 | 2.1 - 2.7 | 0.20 | 0.1 - 0.3 | 29.2 | 96.7 |
| >18 | 80.80 | 72.8 - 87.3 | 68.03 | 64.5 - 71.4 | 2.53 | 2.2 - 2.9 | 0.28 | 0.2 - 0.4 | 30.1 | 95.4 |
| >19 | 80.00 | 71.9 - 86.6 | 69.67 | 66.2 - 73.0 | 2.64 | 2.3 - 3.0 | 0.29 | 0.2 - 0.4 | 31.1 | 95.3 |
| >20 | 75.20 | 66.7 - 82.5 | 74.45 | 71.1 - 77.6 | 2.94 | 2.5 - 3.5 | 0.33 | 0.2 - 0.5 | 33.5 | 94.6 |
| >21 | 72.80 | 64.1 - 80.4 | 75.55 | 72.3 - 78.6 | 2.98 | 2.5 - 3.5 | 0.36 | 0.3 - 0.5 | 33.7 | 94.2 |
| >22 | 68.00 | 59.1 - 76.1 | 78.96 | 75.8 - 81.9 | 3.23 | 2.7 - 3.9 | 0.41 | 0.3 - 0.5 | 35.6 | 93.5 |
| >23 | 63.20 | 54.1 - 71.6 | 81.28 | 78.3 - 84.0 | 3.38 | 2.8 - 4.1 | 0.45 | 0.4 - 0.6 | 36.6 | 92.8 |
| >24 | 60.80 | 51.7 - 69.4 | 83.20 | 80.3 - 85.8 | 3.62 | 2.9 - 4.5 | 0.47 | 0.4 - 0.6 | 38.2 | 92.6 |
| >25 | 57.60 | 48.4 - 66.4 | 84.70 | 81.9 - 87.2 | 3.76 | 3.0 - 4.7 | 0.50 | 0.4 - 0.6 | 39.1 | 92.1 |
| >26 | 56.80 | 47.6 - 65.6 | 85.52 | 82.8 - 88.0 | 3.92 | 3.1 - 5.0 | 0.51 | 0.4 - 0.6 | 40.1 | 92.1 |
| >27 | 52.80 | 43.7 - 61.8 | 87.84 | 85.3 - 90.1 | 4.34 | 3.4 - 5.6 | 0.54 | 0.4 - 0.6 | 42.6 | 91.6 |
| >28 | 49.60 | 40.5 - 58.7 | 88.93 | 86.4 - 91.1 | 4.48 | 3.4 - 5.9 | 0.57 | 0.5 - 0.7 | 43.4 | 91.2 |
| >29 | 42.40 | 33.6 - 51.6 | 90.85 | 88.5 - 92.8 | 4.63 | 3.4 - 6.3 | 0.63 | 0.5 - 0.7 | 44.2 | 90.2 |
| >30 | 40.00 | 31.3 - 49.1 | 91.80 | 89.6 - 93.7 | 4.88 | 3.5 - 6.7 | 0.65 | 0.6 - 0.8 | 45.5 | 90.0 |
| >31 | 37.60 | 29.1 - 46.7 | 93.03 | 90.9 - 94.8 | 5.40 | 3.8 - 7.6 | 0.67 | 0.6 - 0.8 | 48.0 | 89.7 |
| >32 | 36.00 | 27.6 - 45.1 | 93.85 | 91.9 - 95.5 | 5.86 | 4.1 - 8.5 | 0.68 | 0.6 - 0.8 | 50.0 | 89.6 |
| >33 | 32.00 | 23.9 - 40.9 | 94.40 | 92.5 - 96.0 | 5.71 | 3.9 - 8.5 | 0.72 | 0.6 - 0.8 | 49.4 | 89.0 |
| >34 | 27.20 | 19.6 - 35.9 | 95.90 | 94.2 - 97.2 | 6.64 | 4.2 - 10.4 | 0.76 | 0.7 - 0.8 | 53.1 | 88.5 |
| >35 | 24.00 | 16.8 - 32.5 | 96.31 | 94.7 - 97.6 | 6.51 | 4.0 - 10.6 | 0.79 | 0.7 - 0.9 | 52.6 | 88.1 |
| >36 | 20.80 | 14.1 - 29.0 | 96.99 | 95.5 - 98.1 | 6.92 | 4.1 - 11.8 | 0.82 | 0.7 - 0.9 | 54.2 | 87.8 |
| >37 | 19.20 | 12.7 - 27.2 | 97.68 | 96.3 - 98.6 | 8.27 | 4.6 - 14.9 | 0.83 | 0.8 - 0.9 | 58.5 | 87.6 |
| >38 | 16.80 | 10.7 - 24.5 | 98.09 | 96.8 - 99.0 | 8.78 | 4.6 - 16.8 | 0.85 | 0.8 - 0.9 | 60.0 | 87.3 |
| >39 | 14.40 | 8.8 - 21.8 | 98.36 | 97.2 - 99.2 | 8.78 | 4.3 - 17.8 | 0.87 | 0.8 - 0.9 | 60.0 | 87.1 |
| >40 | 12.80 | 7.5 - 20.0 | 98.63 | 97.5 - 99.3 | 9.37 | 4.4 - 20.2 | 0.88 | 0.8 - 0.9 | 61.5 | 86.9 |
| >41 | 11.20 | 6.3 - 18.1 | 99.18 | 98.2 - 99.7 | 13.66 | 5.4 - 34.9 | 0.90 | 0.8 - 1.0 | 70.0 | 86.7 |
| >43 | 8.00 | 3.9 - 14.2 | 99.45 | 98.6 - 99.9 | 14.64 | 4.7 - 46.0 | 0.93 | 0.9 - 1.0 | 71.4 | 86.4 |
| >44 | 5.60 | 2.3 - 11.2 | 99.45 | 98.6 - 99.9 | 10.25 | 3.0 - 34.5 | 0.95 | 0.9 - 1.0 | 63.6 | 86.1 |
| >45 | 4.00 | 1.3 - 9.1 | 100.00 | 99.5 - 100.0 |  |  | 0.96 | 0.9 - 1.0 | 100.0 | 85.9 |
| >48 | 2.40 | 0.5 - 6.9 | 100.00 | 99.5 - 100.0 |  |  | 0.98 | 0.9 - 1.0 | 100.0 | 85.7 |
| >53 | 1.60 | 0.2 - 5.7 | 100.00 | 99.5 - 100.0 |  |  | 0.98 | 1.0 - 1.0 | 100.0 | 85.6 |
| >57 | 0.00 | 0.0 - 2.9 | 100.00 | 99.5 - 100.0 |  |  | 1.00 | 1.0 - 1.0 |  | 85.4 |

**Table S5: DNR classification of >17 cutoff value.**

|  |  | **Actual DNR Status** | |  |
| --- | --- | --- | --- | --- |
|  |  | **+** | **-** | **Total** |
| **ePOS > 17 classification** | **+** | **TP: 109** | **FP: 264** | **373** |
|  | **-** | **FN: 16** | **TN: 468** | **484** |
|  | **Total** | **125** | **732** | **857** |
